# Supplementary material for: Regulation of a Novel Splice Variant of Early Growth Response 4 (EGR4-S) by HER+ Signalling and HSF1 in Breast Cancer
Source: Cancers (Basel). 2022 Mar 18;14(6):1567. doi: 10.3390/cancers14061567 (PMC8946690; doi:10.3390/cancers14061567)
Supplement: Supplementary file 1 [file cancers-14-01567-s001.zip › cancers-1517940. supplementary.pdf]

cancers

MDPI

## Supplementary Materials:

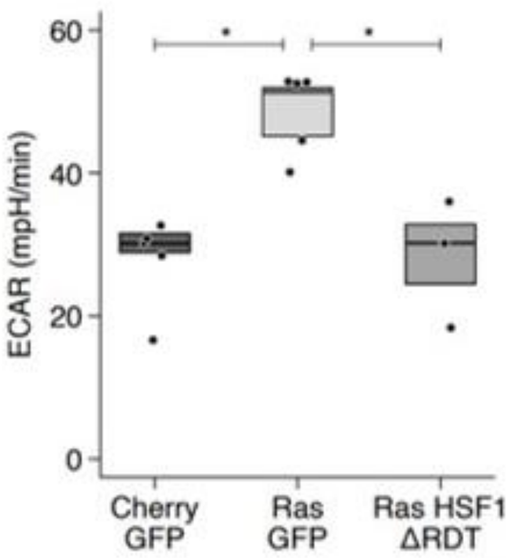


**Figure S1.** Glycolytic capacity of transformed MCF10A cells. Analysis of cell glycolytic capacity in oncogenically transformed MCF10A cells. Significantly increased glycolytic capacity was observed in transformed cells (Ras GFP, * $p < 0.05$) compared to non-transformed cells (Cherry GFP). Significantly lower glycolytic capacity was observed in transformed cells with elevated HSF1 (Ras HSF1 ΔRDT, * $p < 0.05$).

A

| Analysis | EGR4-1 | EGR4-2 | EGR4-S |
|---|---|---|---|
| Length | 589 aa | 486 aa | 382aa |
| Predicted Molecular Weight | 61619.01 m.w. | 50795.24 m.w | 40012.94 m.w. |
| 1 microgram = | 16.229 pMoles | 19.687 pMoles | 24.992pMoles |
| Molar Extinction coefficient | 38080 | 30630 | 21900 |
| 1 A[280] corr. to | 1.62 mg/ml | 1.66 mg/ml | 1.83 mg/ml |
| A[280] of 1mg/ml | 0.62 AU | 0.60 AU | 0.55 AU |
| Isoelectric Point | 8.75 | 6.21 | 8.66 |
| Charge at pH 7 | 10.77 | -4.29 | 6.46 |
| Charged amino acids (RKHYCDE) | 164 (34.51% w/w; 27.84% freq.) | 131 (32.97% w/w; 26.95% freq.) | 105 (33.80% w/w; 27.49% freq.) |
| Acidic amino acids (DE) | 55 (10.74% w/w; 9.34% freq.) | 49 (11.57% w/w; 10.08% freq.) | 35 (10.48% w/w; 9.16% freq.) |
| Basic amino acids (KR) | 65 (14.94% w/w; 11.04 freq.) | 44 (12.07% w/w; 9.05% freq.) | 41 (14.28% w/w; 10.73% freq.) |
| Polar amino acids (NCQSTY) | 114 (19.33% w/w; 19.35% freq.) | 100 (20.53% w/w; 20.58% freq.) | 77 (20.16% w/w; 20.16% freq.) |
| Hydrophobic amino acids (AIFWV) | 191 (31.31% w/w; 32.43 freq.) | 164 (33.12% w/w; 33.74% freq.) | 127 (32.44% w/w; 33.25% freq.) |

B

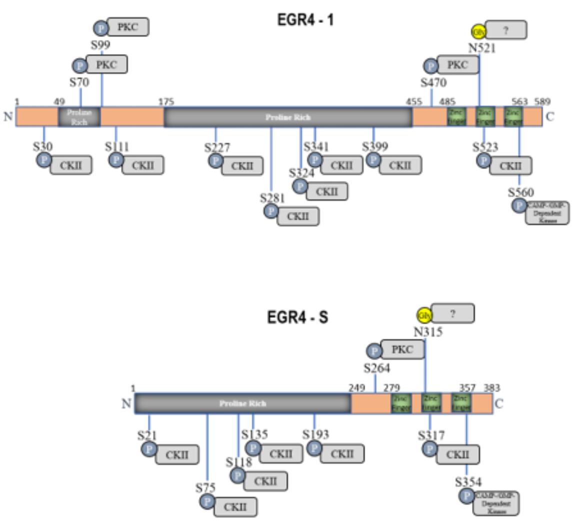


**Figure S2.** Analysis of EGR4 isoforms. **(A)** Biostructural analysis of the 3 possible protein isoforms of EGR4 reveals 2 potential proteins (EGR4-1 and EGR4-S) and one unlikely possibility (EGR4-2) **(B)** Schematic diagrams of (top) the longer EGR4-1 isoform showing two proline-rich regions and multiple phosphorylation sites and (bottom) the shorter/truncated EGR4-S isoform with one single proline-rich region and less phosphorylation sites.

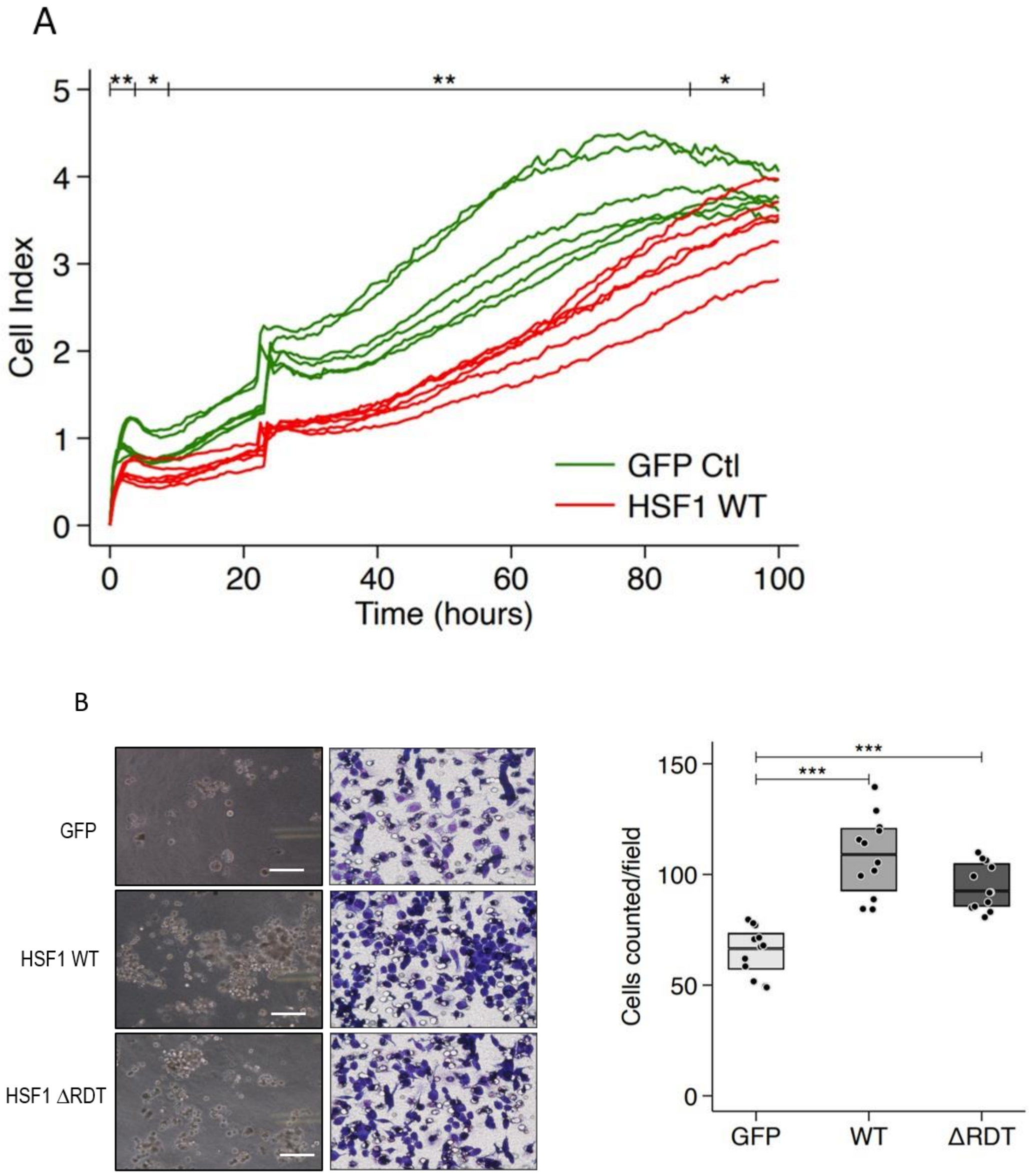


**Figure S3.** Effect of altered HSF1 on cancer cell characteristics. **(A)** Proliferation Assay showing the effect of elevated HSF1 expression (HSF1 WT) on HER2+ cell proliferation over time (* $p$<0.05, ** $p$<0.01) – note cell numbers were normalised from the start of all experiments to show proliferation rate **(B)** 3D culture of HER2+ cells grown from single cell origin (left panels) and migration assay results (right panels) from cells with elevated HSF1 (WT, ΔRDT *** $p$<0.001) compared to control (GFP).
