# Supplementary material for: Regulation of a Novel Splice Variant of Early Growth Response 4 (EGR4-S) by HER+ Signalling and HSF1 in Breast Cancer
Source: Cancers (Basel). 2022 Mar 18;14(6):1567. doi: 10.3390/cancers14061567 (PMC8946690; doi:10.3390/cancers14061567)
Supplement: Supplementary file 1 [file cancers-14-01567-s001.zip › cancers-1517940.WB figurespdf.pdf]

A

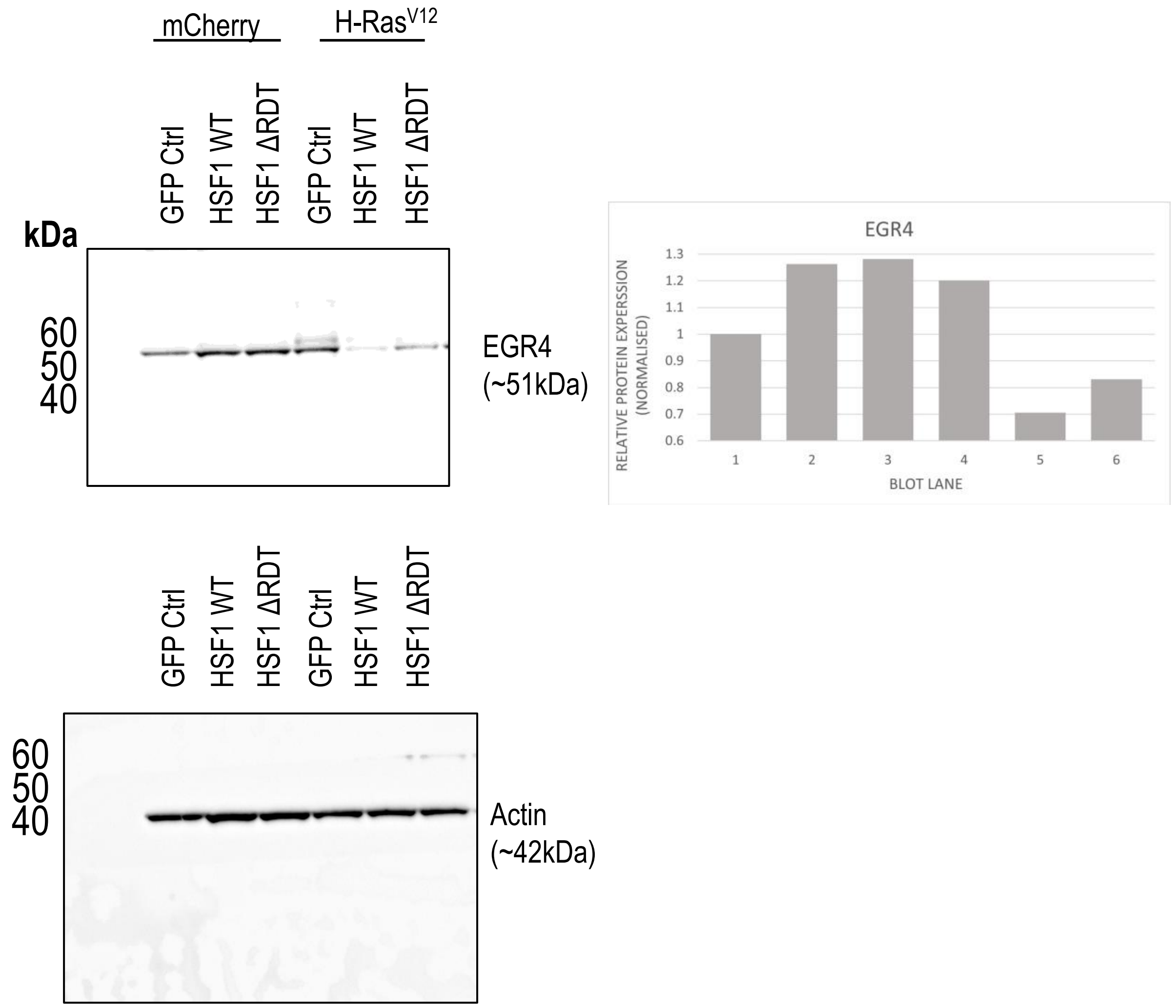

Figure 1A: Association of EGR4 with HSF1 and HER2/HER1 expression in breast cell lines

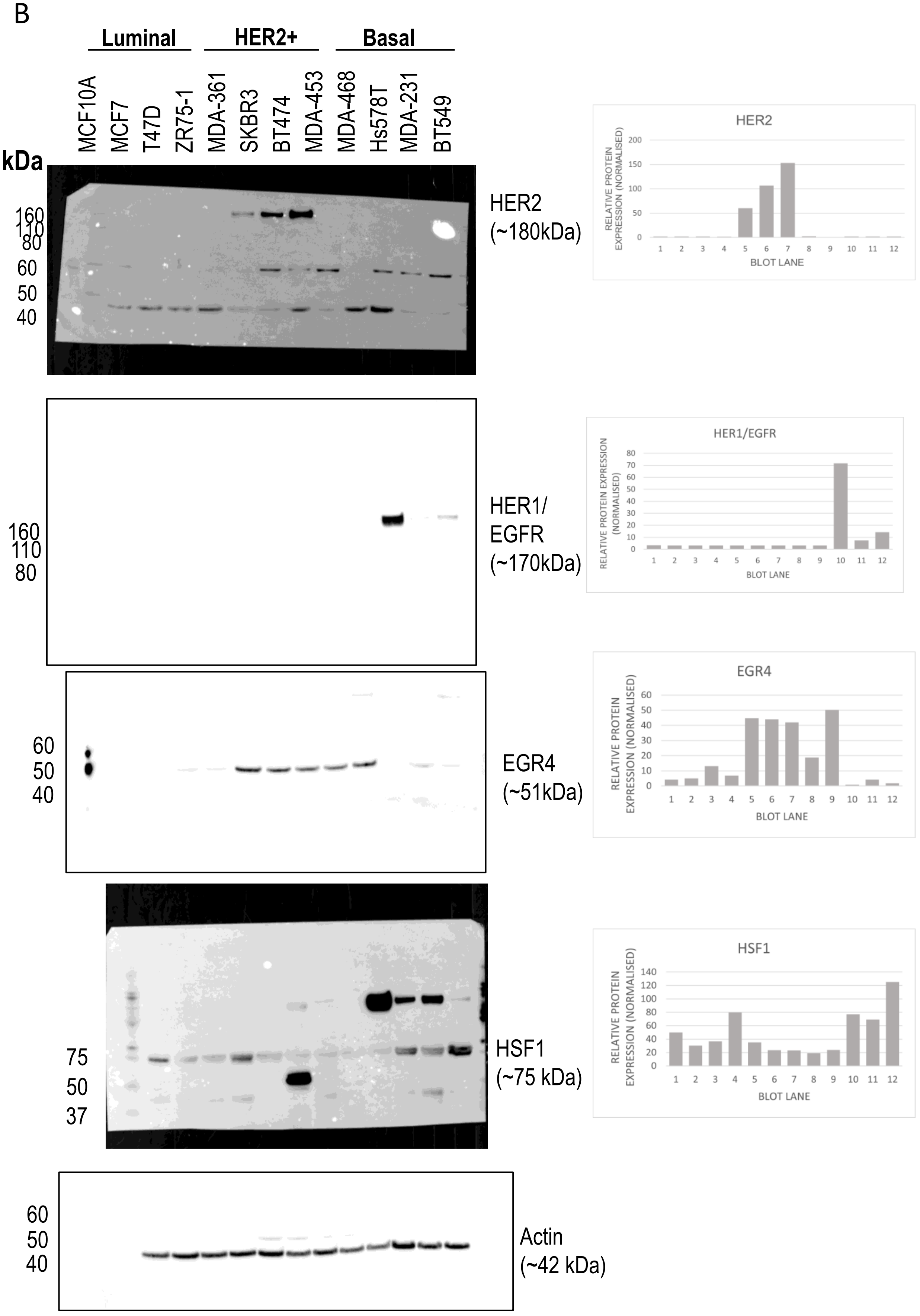

Figure 1B: Association of EGR4 with HSF1 and HER2/HER1 expression in breast cell lines

C

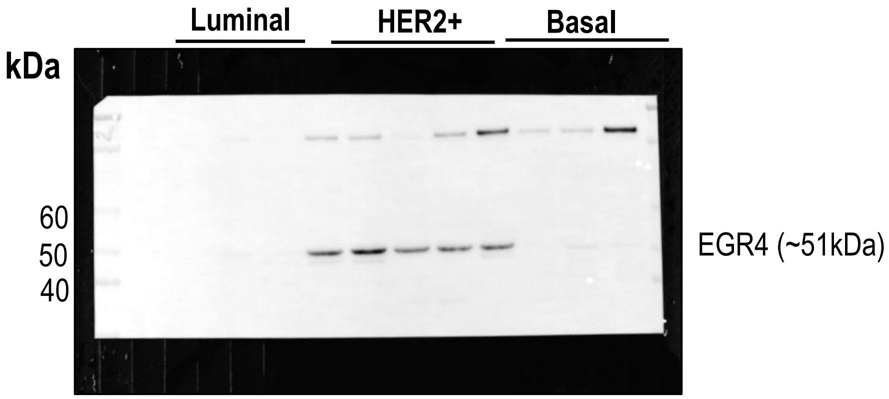

Figure 1C: Association of EGR4 with HSF1 and HER2/HER1 expression in breast cell lines

C

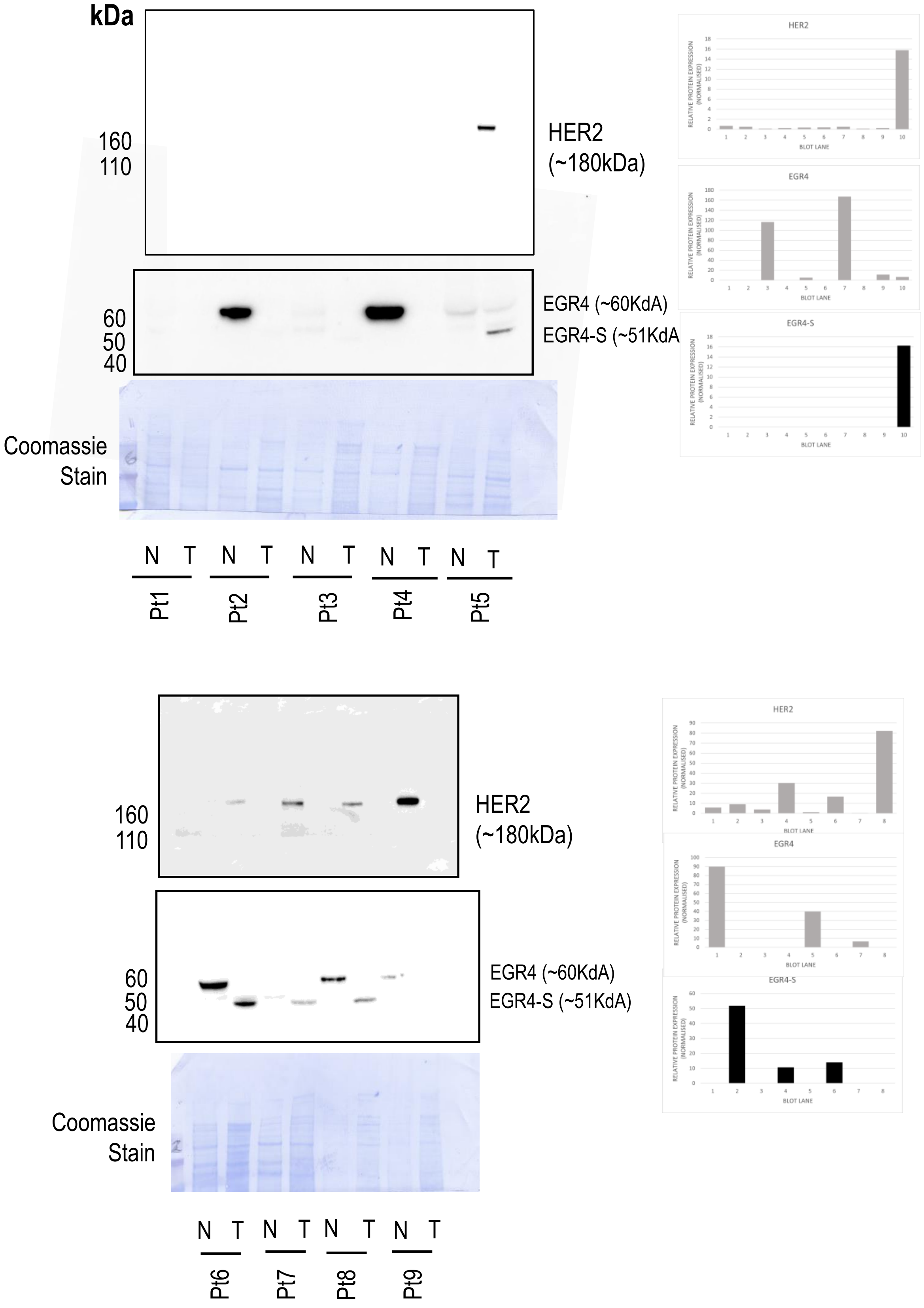

Figure 2C: Structure of EGR4 gene and splice variant expression in cells and tumour tissue

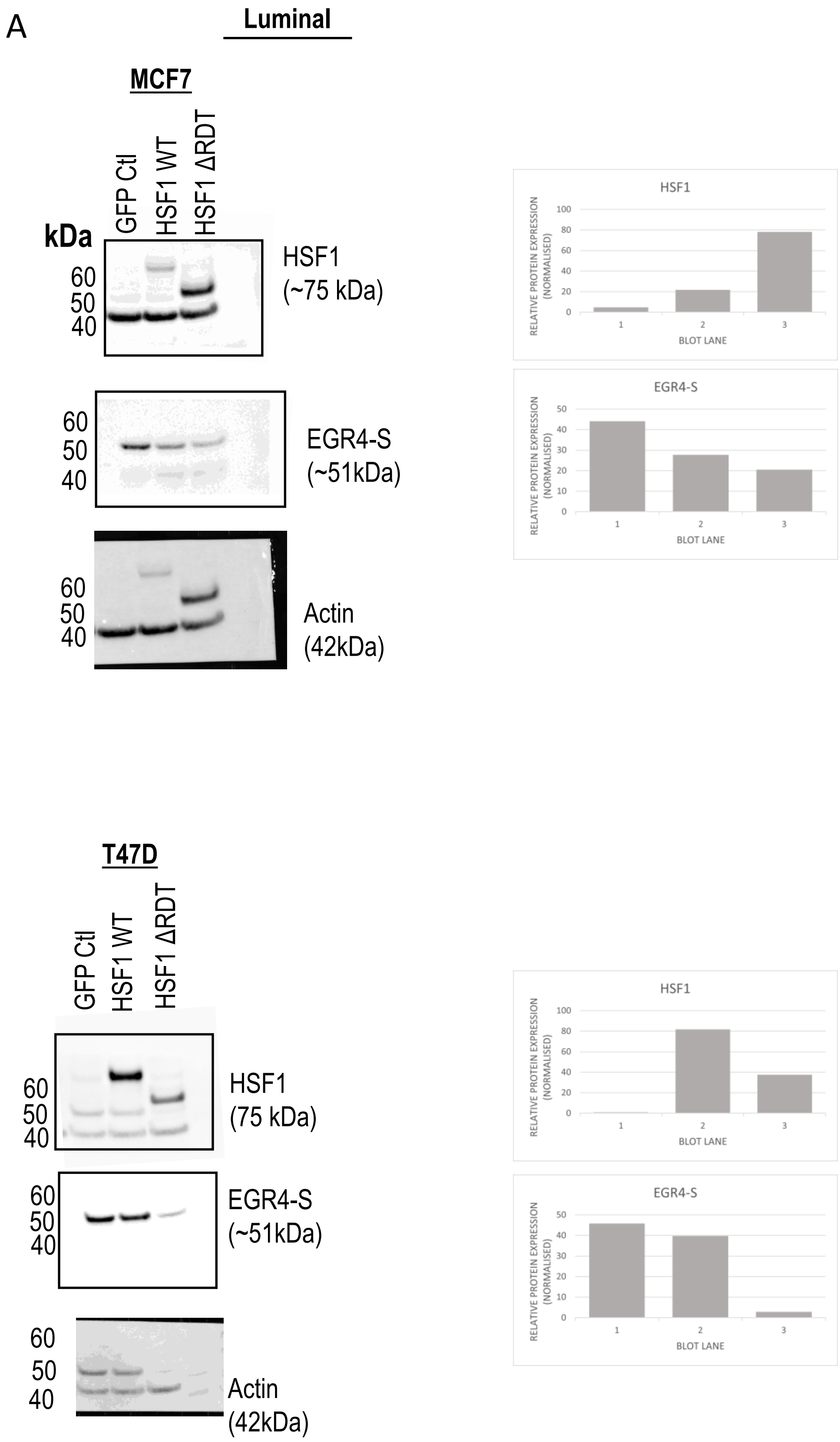

Figure 4A: Inverse association between HSF1 and EGR4-S expression

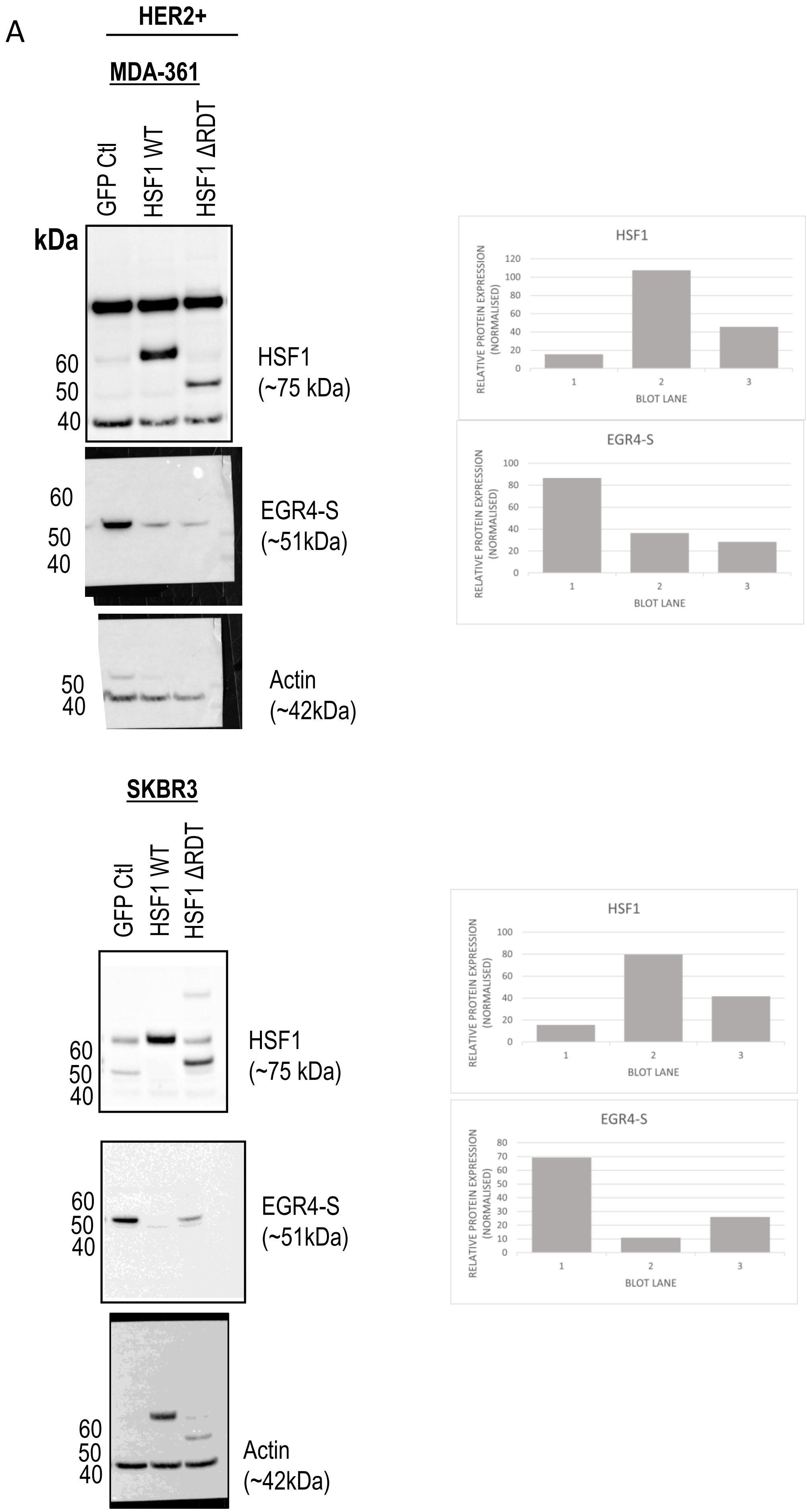

Figure 4A: Inverse association between HSF1 and EGR4-S expression

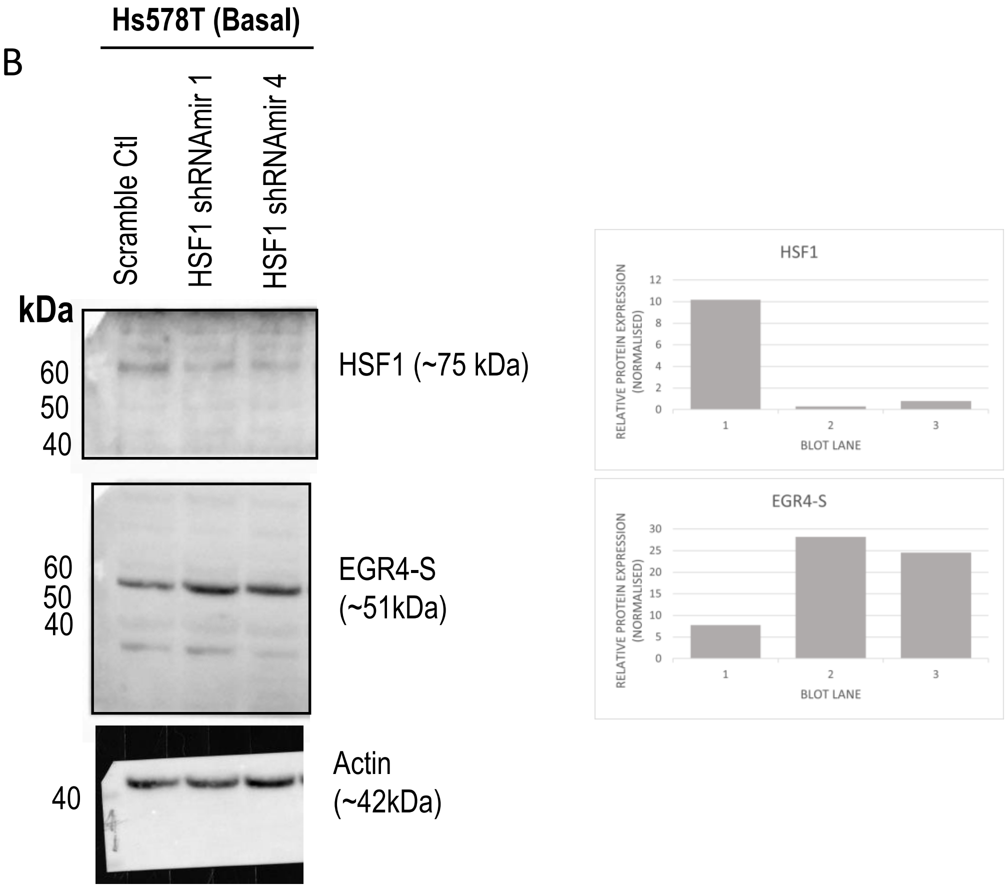

Figure 4B: Inverse association between HSF1 and EGR4-S expression

C

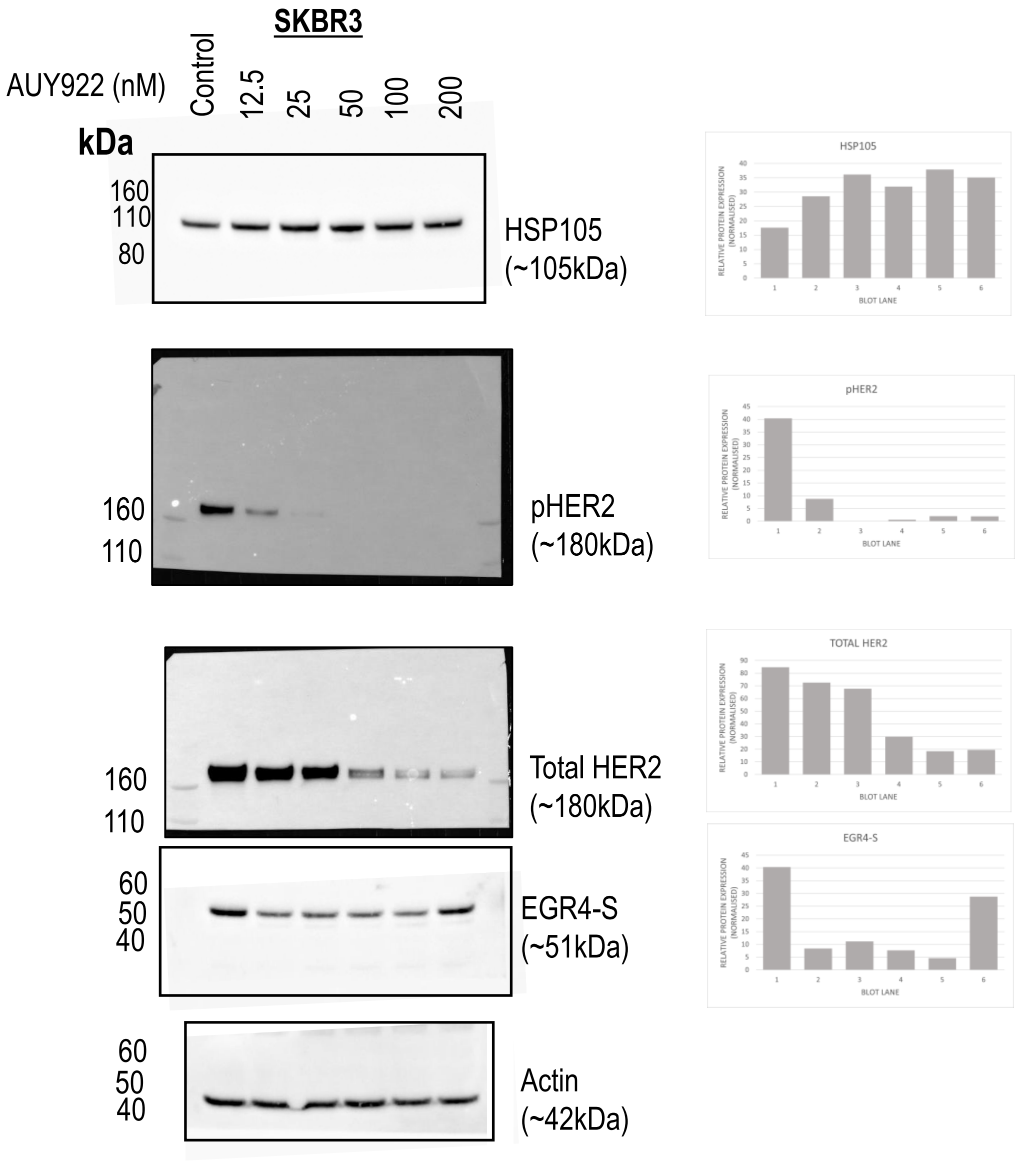

Figure 4C: Inverse association between HSF1 and EGR4-S expression

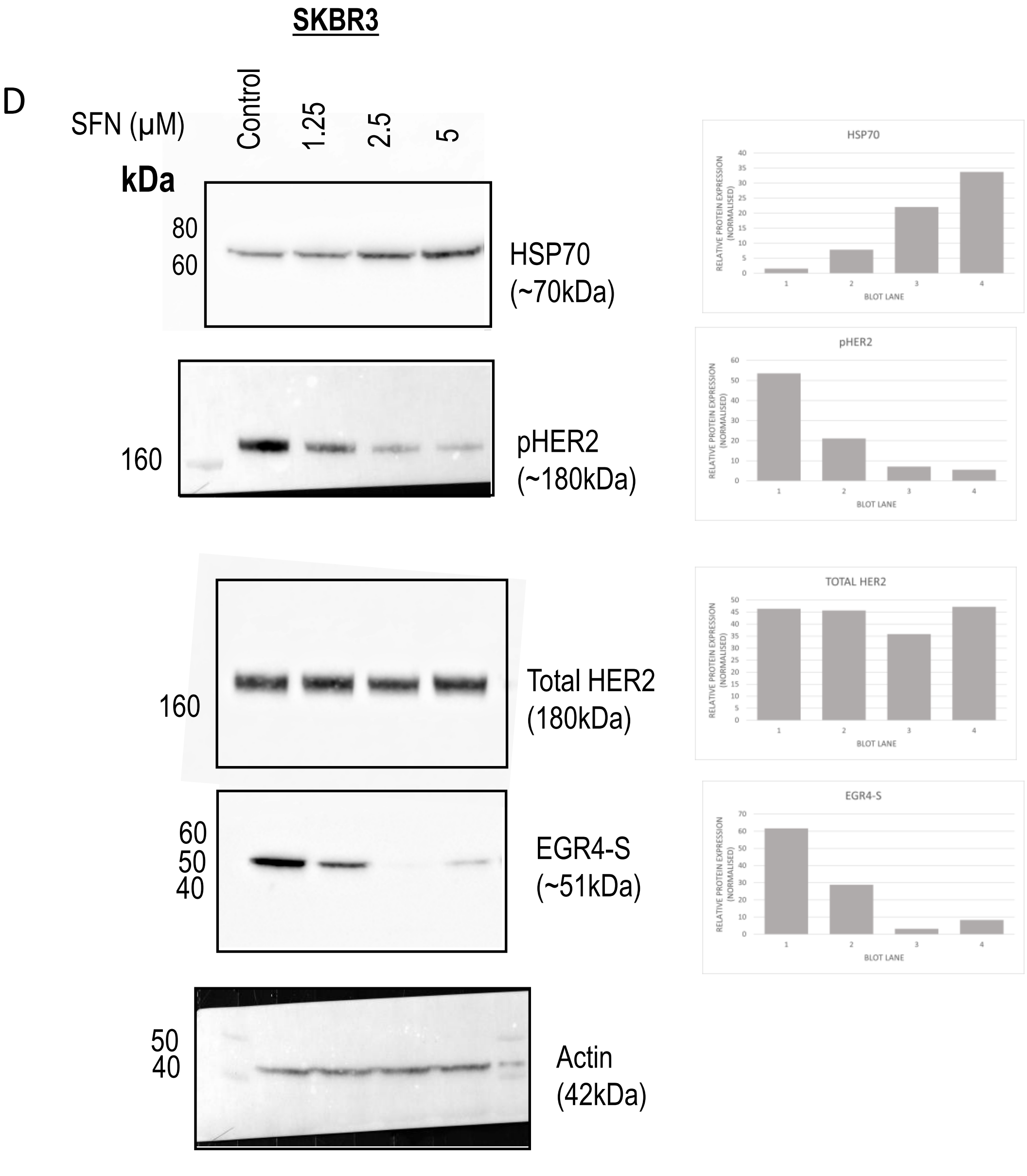

Figure 4C: Inverse association between HSF1 and EGR4-S expression

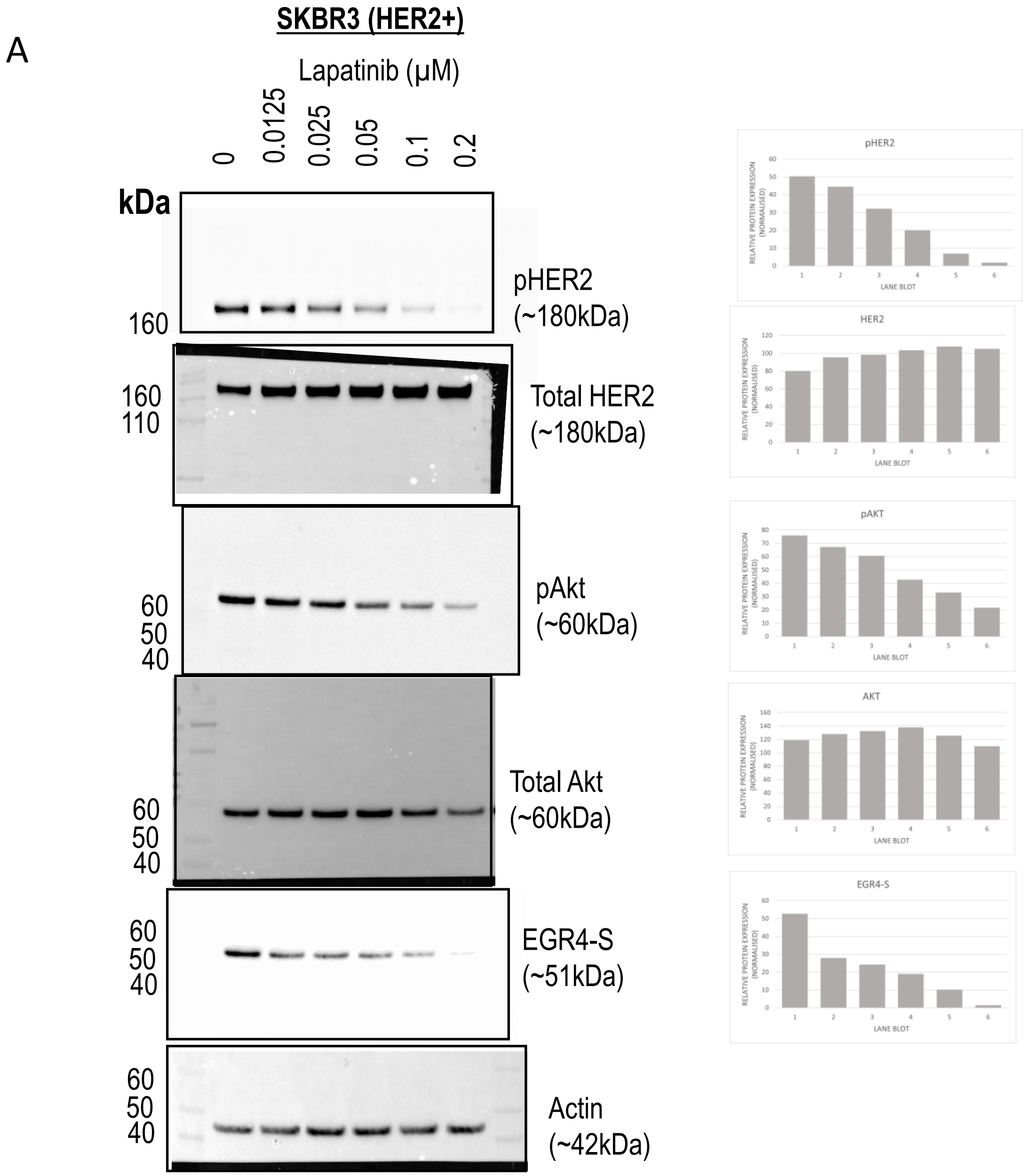

Figure 5A: EGR4-S expression is regulated by HER-pathway targeted drug treatment

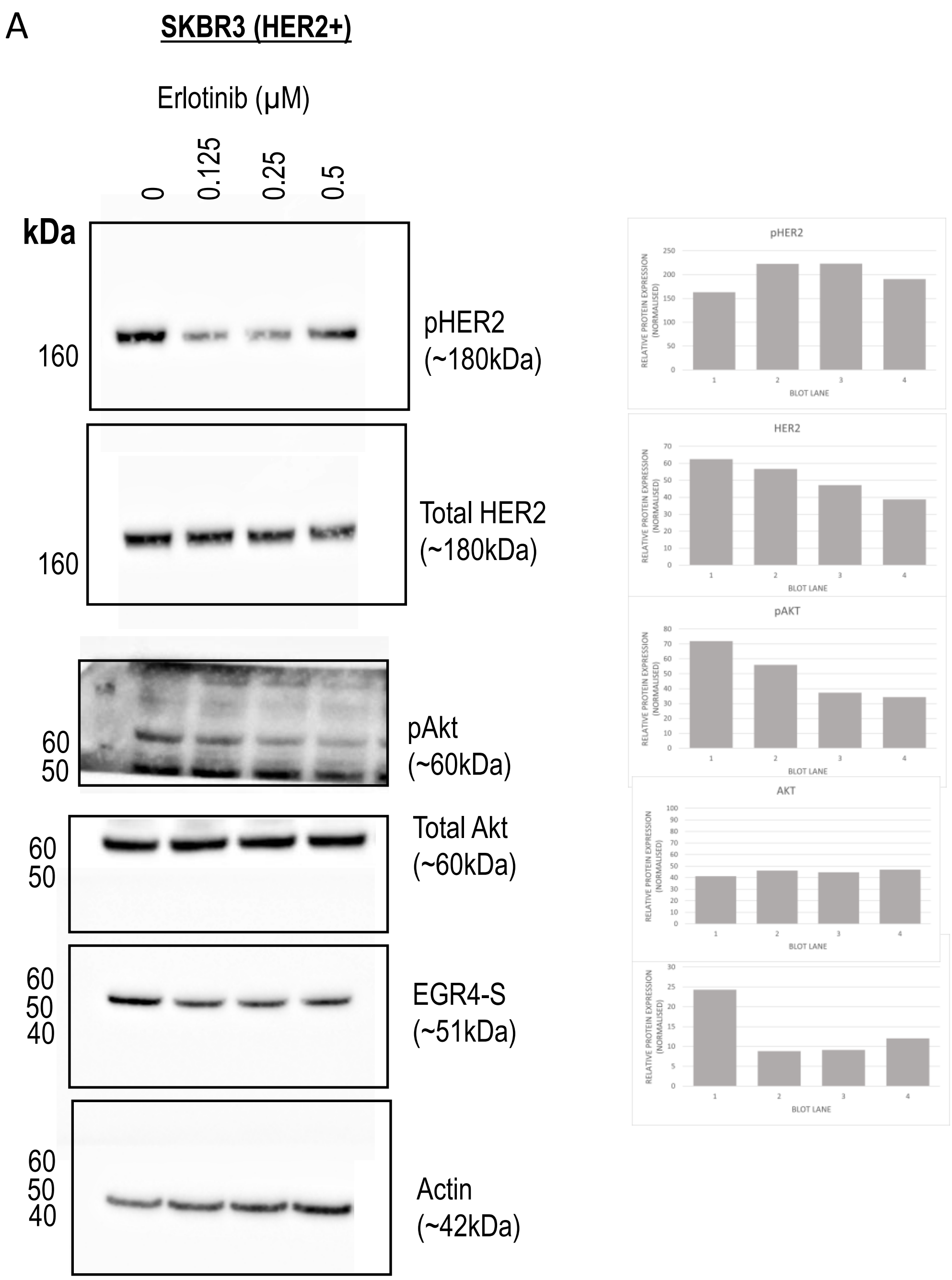

Figure 5A: EGR4-S expression is regulated by HER-pathway targeted drug treatment

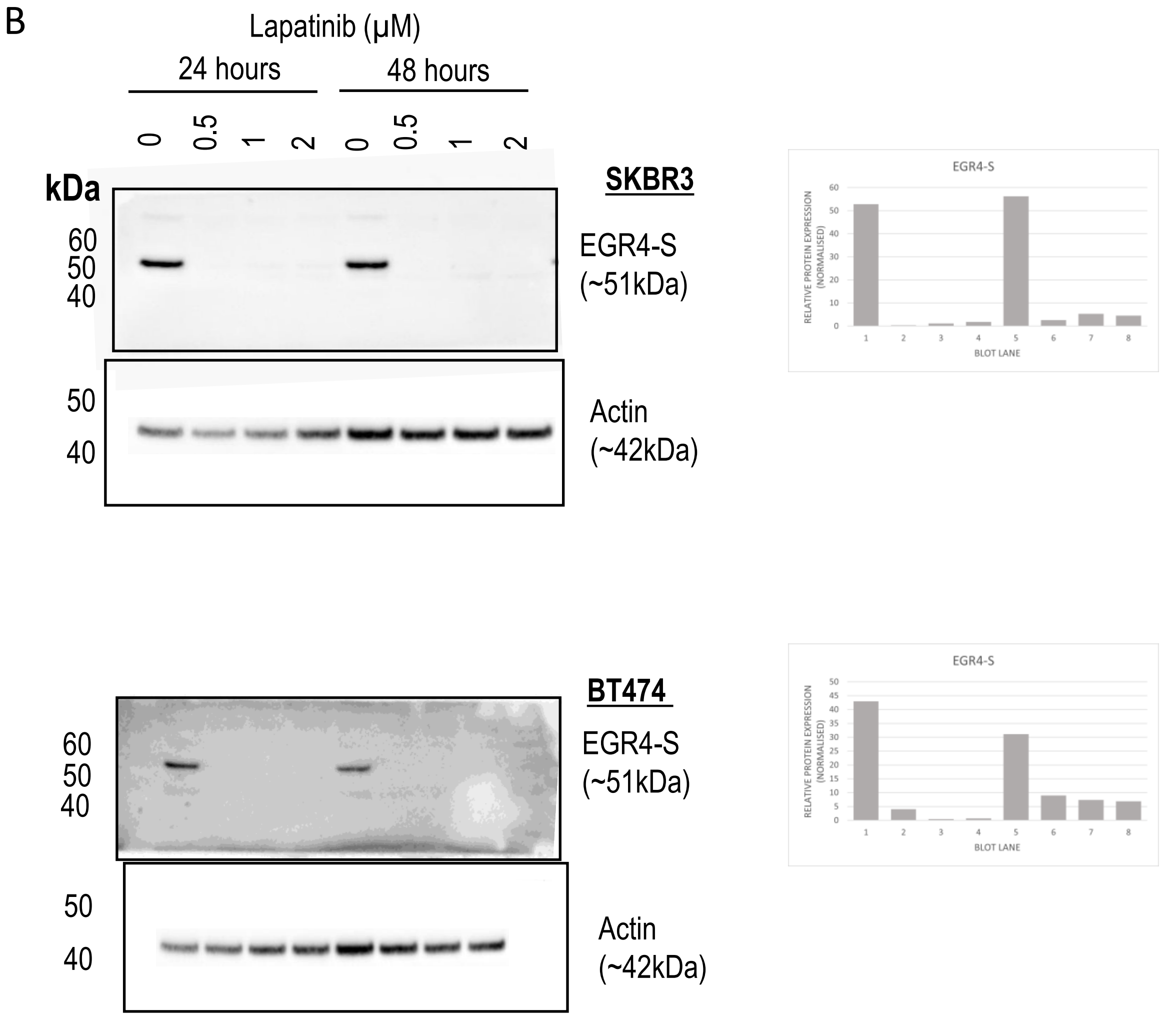

Figure 5B: EGR4-S expression is regulated by HER-pathway targeted drug treatment

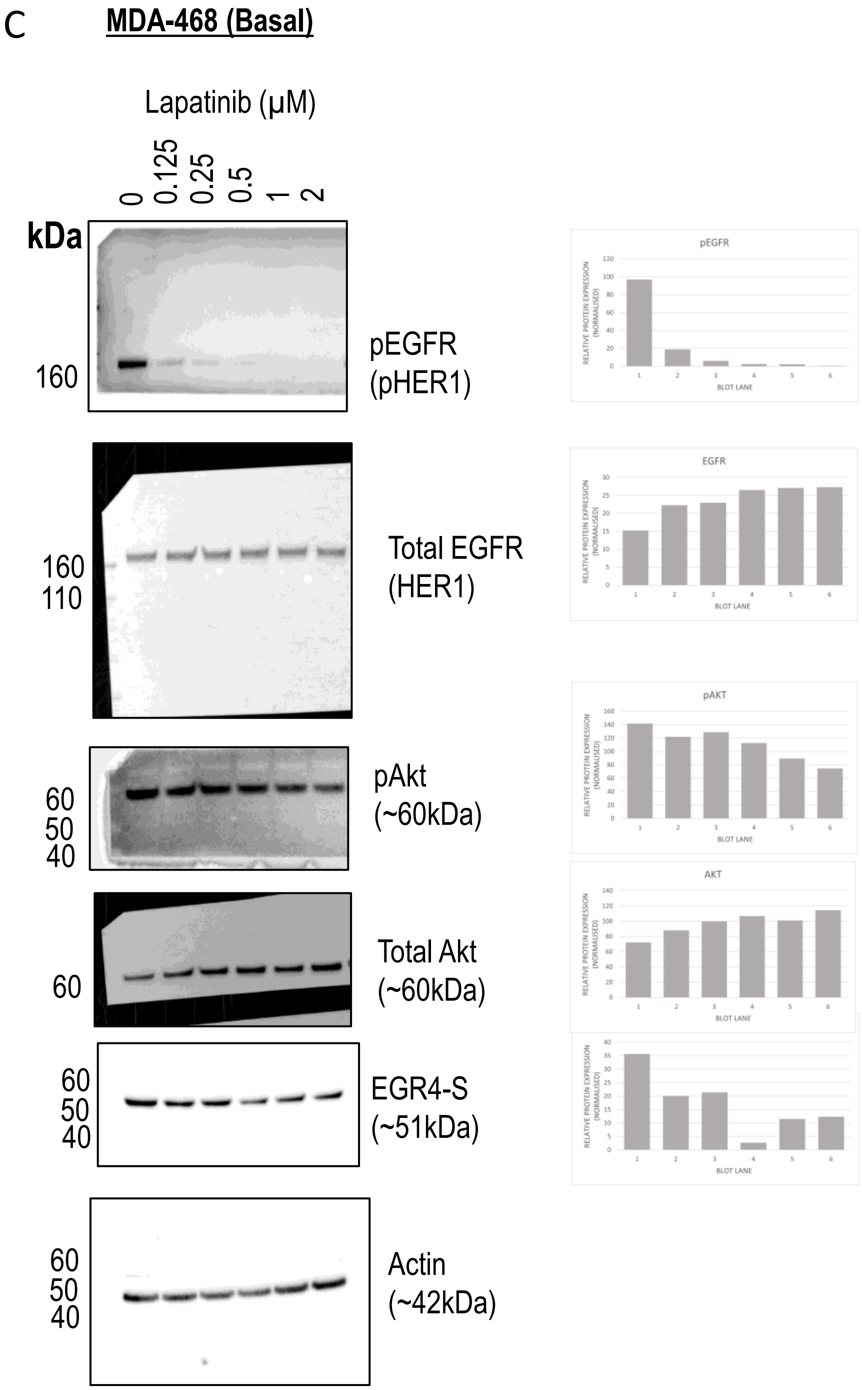

Figure 5C: EGR4-S expression is regulated by HER-pathway targeted drug treatment

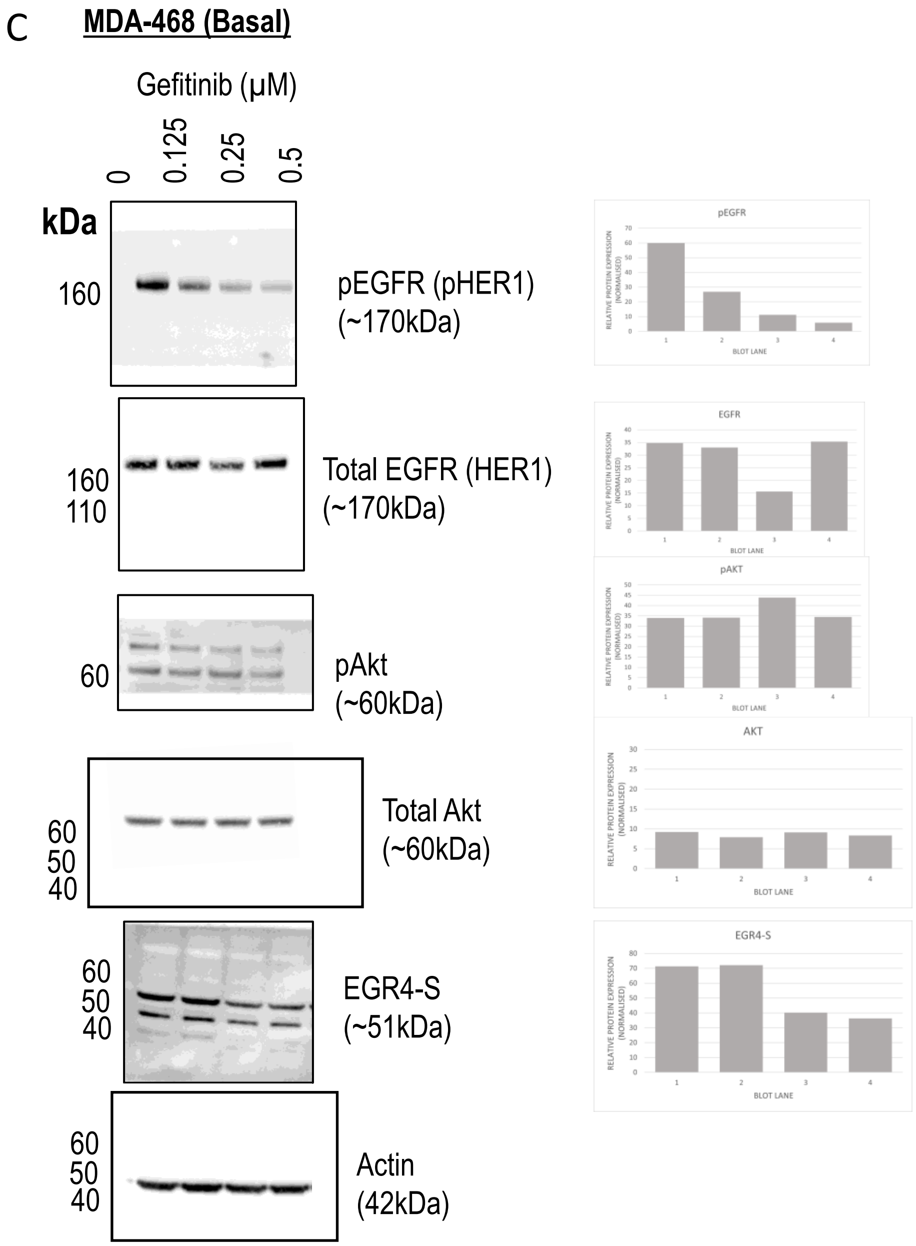

Figure 5C: EGR4-S expression is regulated by HER-pathway targeted drug treatment

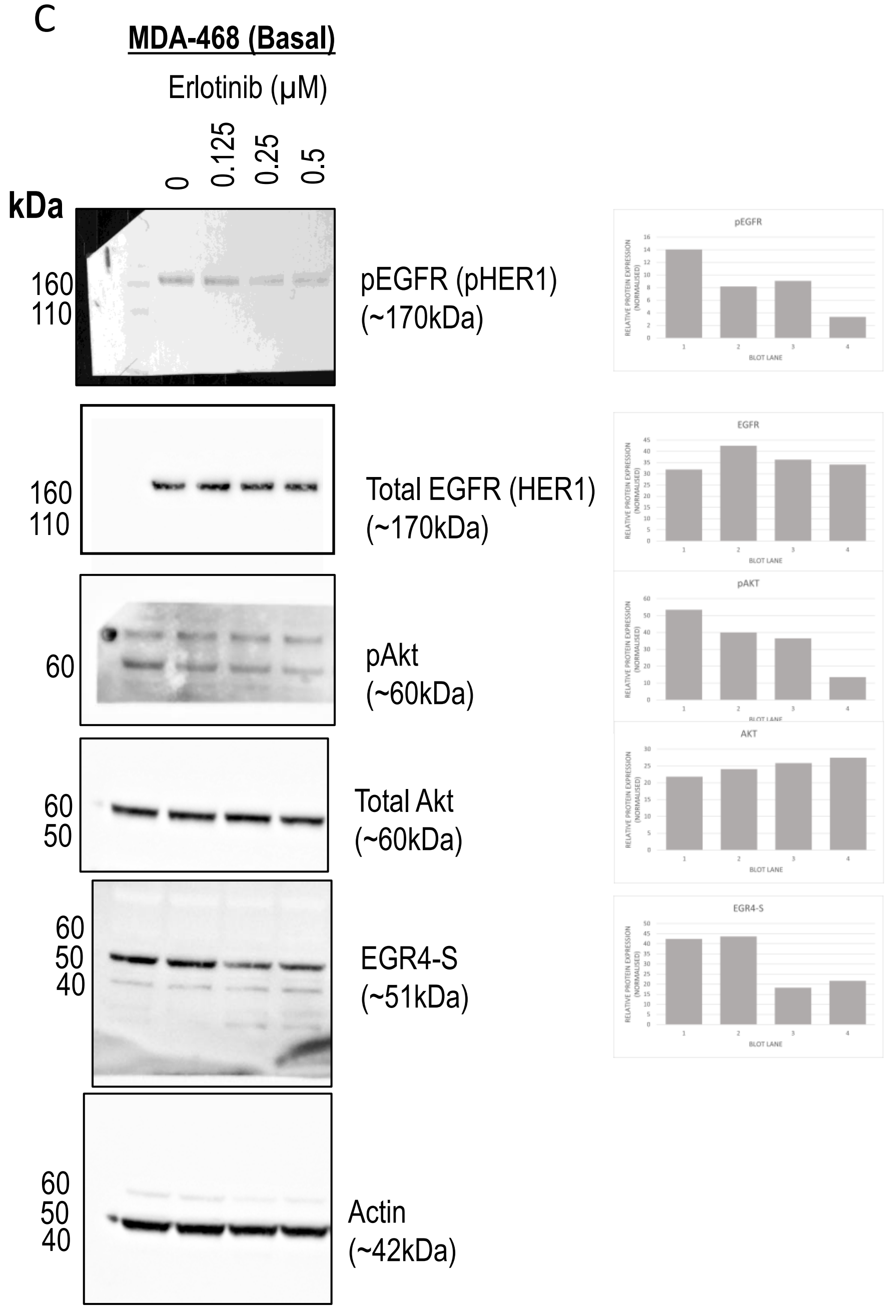

Figure 5C: EGR4-S expression is regulated by HER-pathway targeted drug treatment

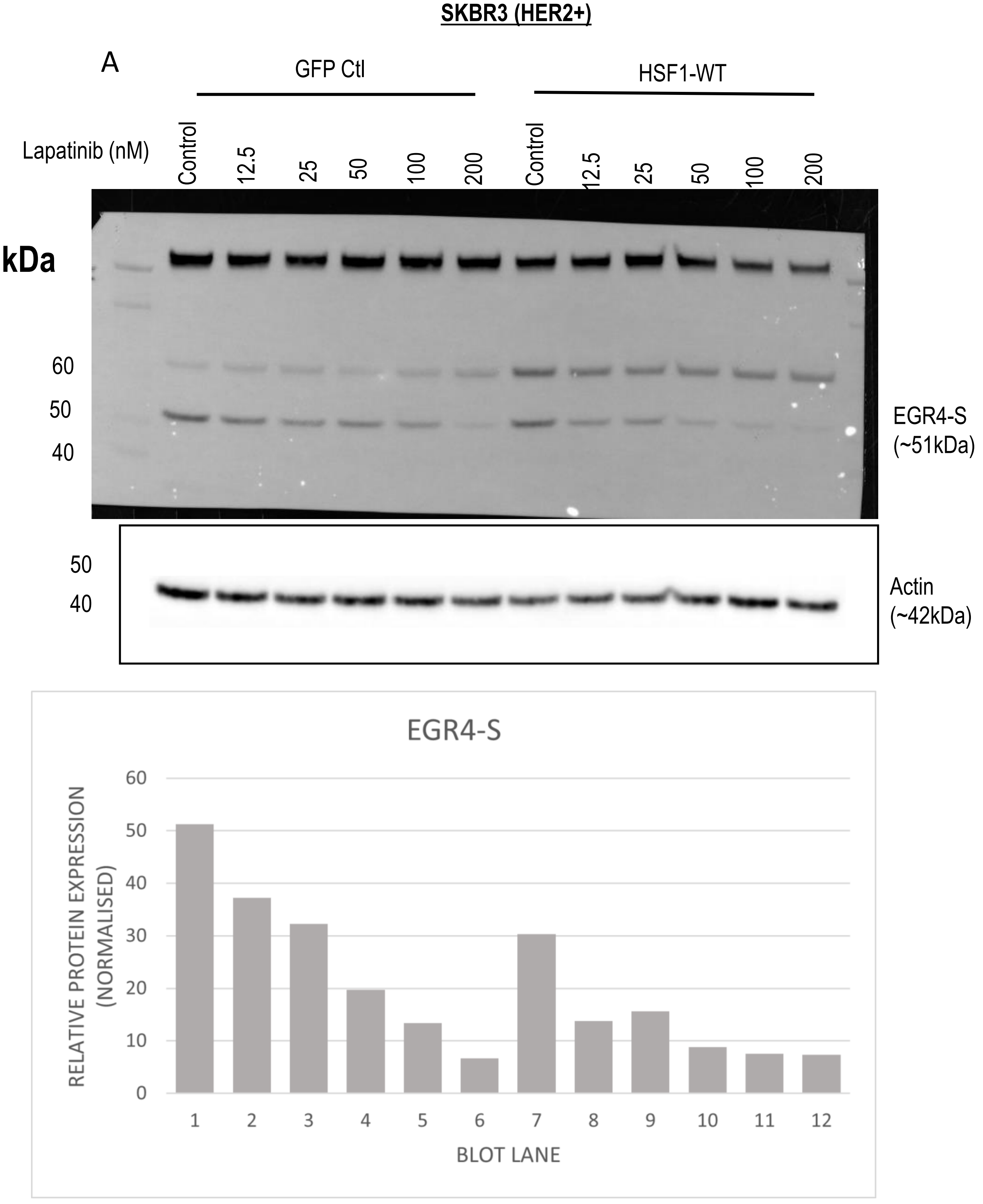

Figure 6: EGR4-S expression is responsive to HER-targeted treatment and affected by HSF1

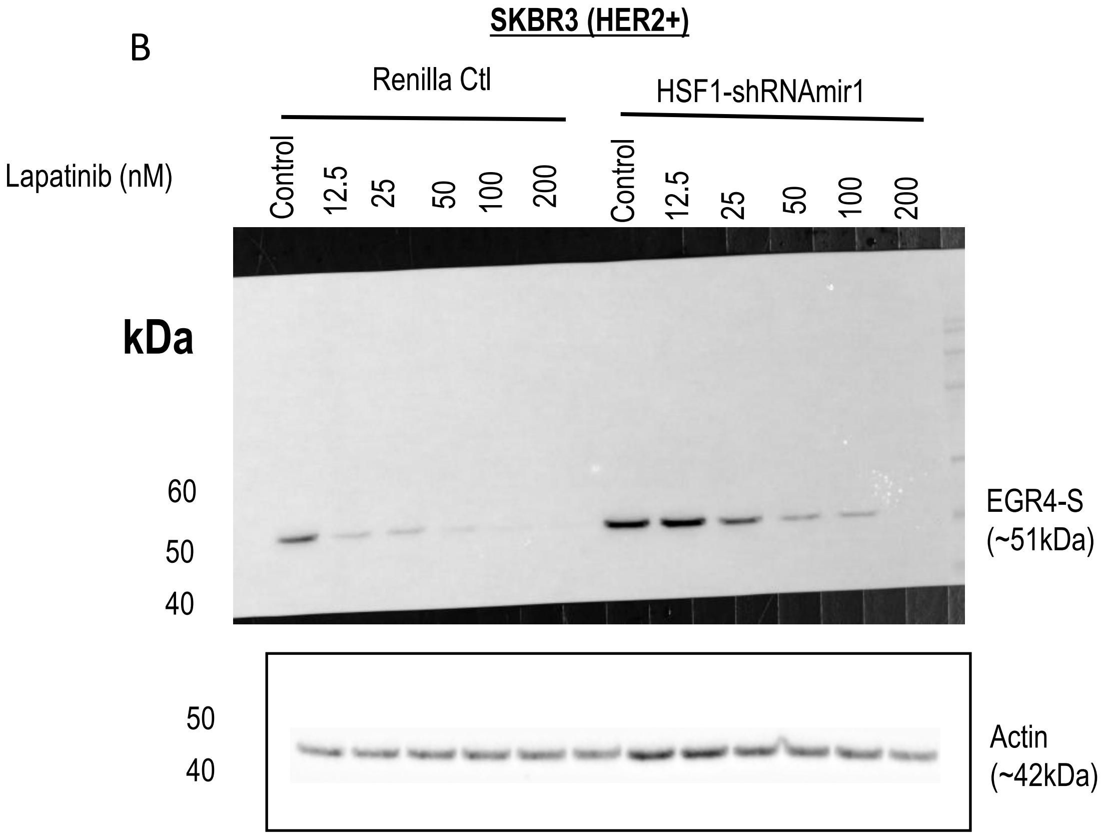

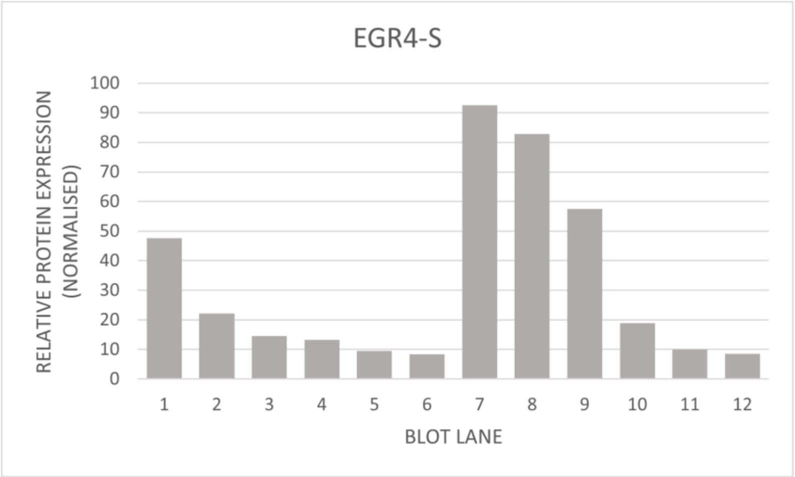

Figure 6: EGR4-S expression is responsive to HER-targeted treatment and affected by HSF1

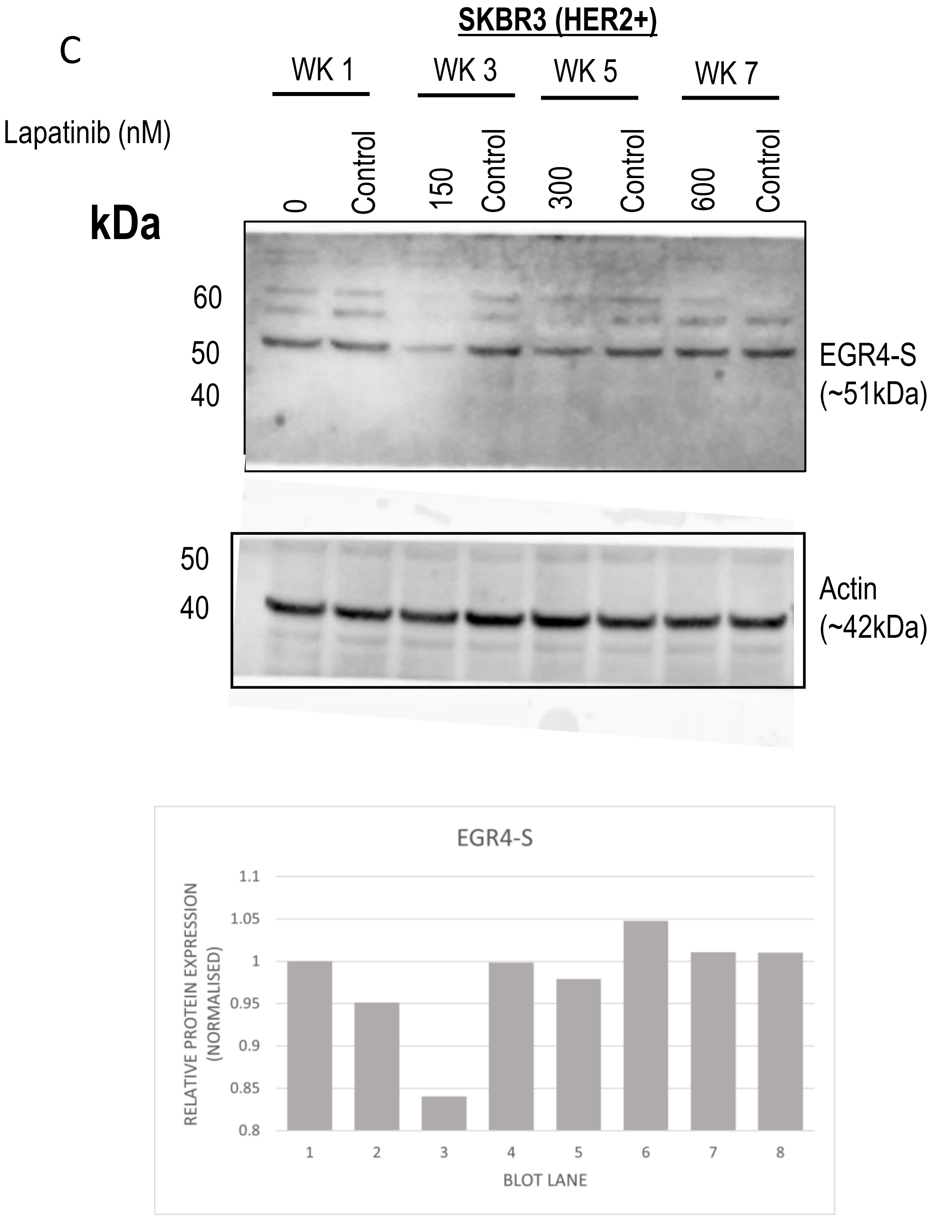

Figure 6: EGR4-S expression is responsive to HER-targeted treatment and affected by HSF1

D

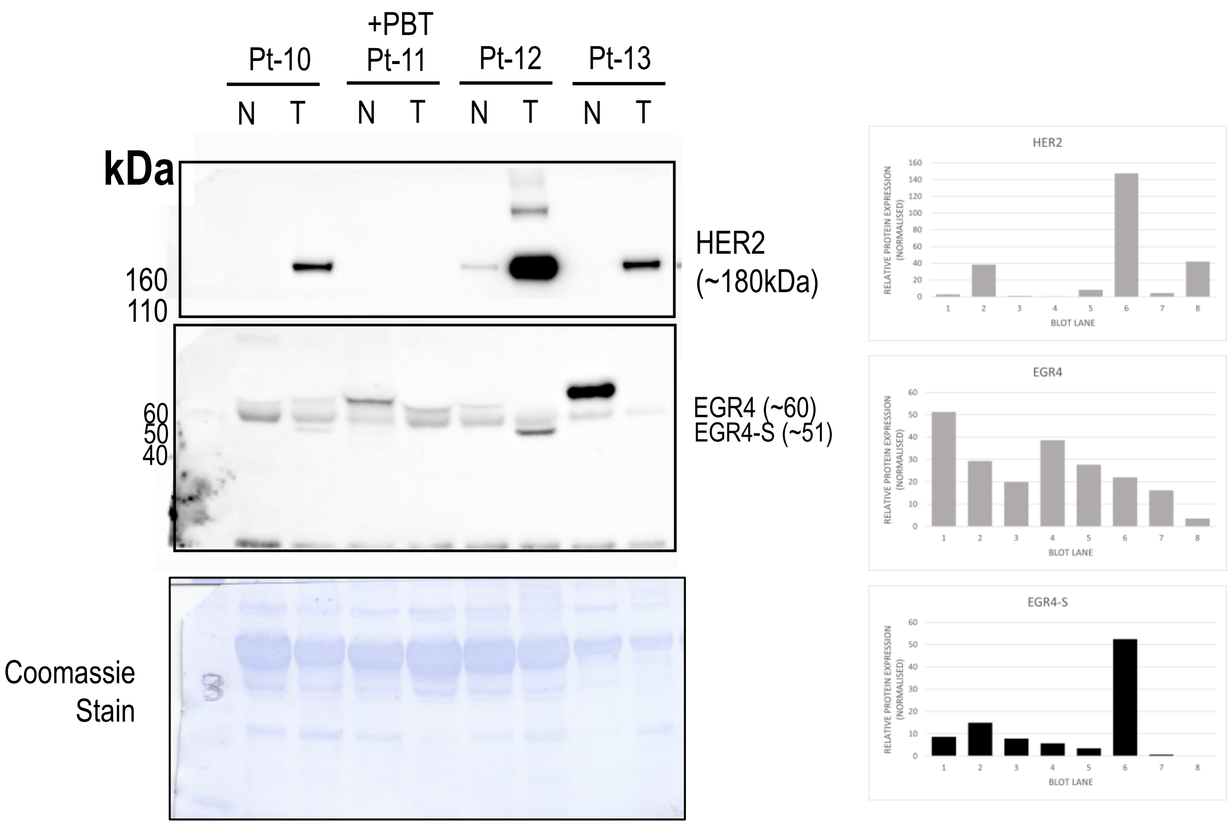

Figure 6: EGR4-S expression is responsive to HER-targeted treatment and affected by HSF1

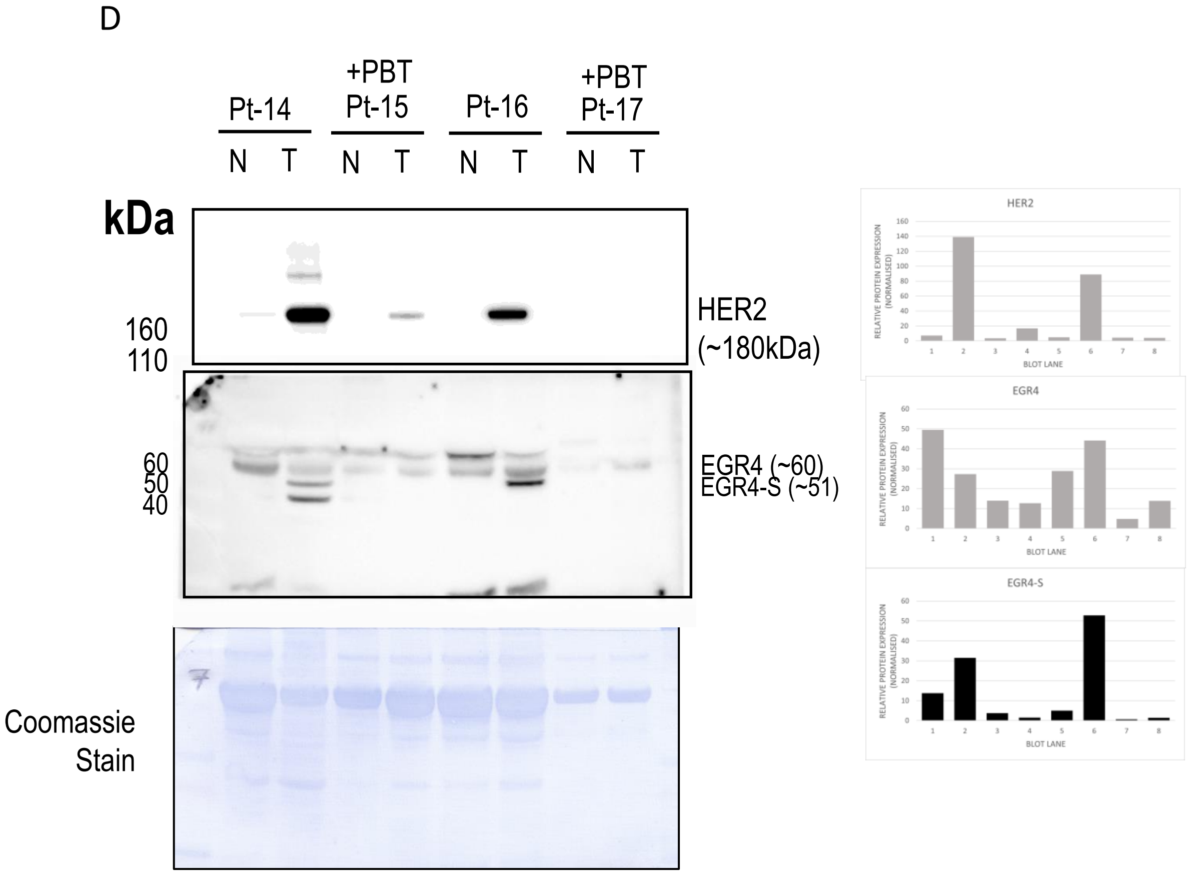

Figure 6: EGR4-S expression is responsive to HER-targeted treatment and affected by HSF1

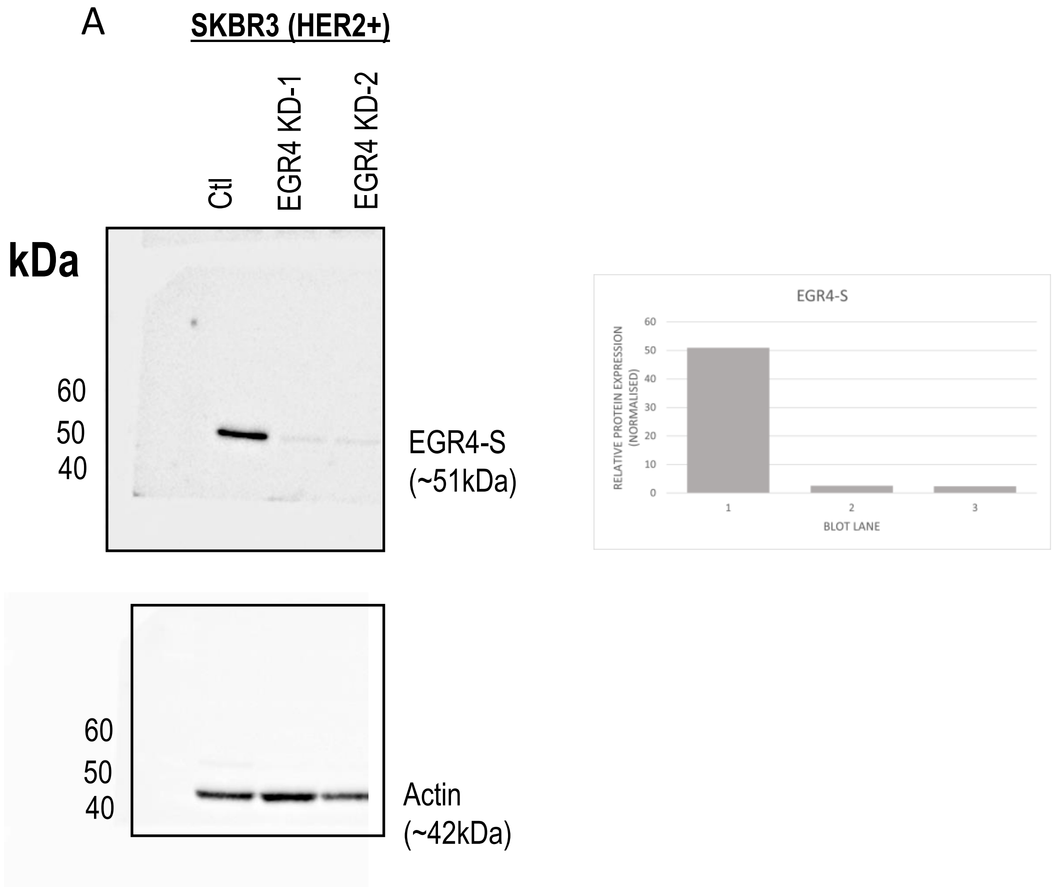

Figure 7: Effect of altered EGR4 expression on cancer cell growth
